# Ferulic acid improves motor function induced by spinal cord injury in rats via inhibiting neuroinflammation and apoptosis

**DOI:** 10.1590/ACB360705

**Published:** 2021-09-03

**Authors:** Xi Jiang, Xuefeng Yu, Jin Chen, Changfeng Jing, Lexing Xu, Ziwei Chen, Fuhe Liu, Lei Chen

**Affiliations:** 1 MM. Department of Pharmacy - Zhejiang University Mingzhou Hospital and Zhejiang Pharmaceutical College - Ningbo, China.; 2 MM. Department of Pharmacy - Zhejiang Pharmaceutical College - Ningbo, China.; 3 BM. Department of Pharmacy - Zhejiang University Mingzhou Hospital - Ningbo, China.; 4 MM. Department of Pharmacy - Zhejiang University Mingzhou Hospital - Ningbo, China.; 5 PhD. Department of Pharmacy - Zhejiang Pharmaceutical College - Ningbo, China.

**Keywords:** Central Cord Syndrome, Apoptosis, Rats

## Abstract

**Purpose:**

To investigate the effect of ferulic acid (FA) on spinal cord injury (SCI)-induced motor dysfunction and to explore the possible pharmacological mechanisms.

**Methods:**

Adult male Wistar rats were used in our study. SCI was achieved by clipping the spinal cord T9 of the rat by a vascular clip for 2 minutes. The motor function of the rat was evaluated by Basso, Beattie, and Bresnahan scoring method (BBB) and inclined plane test. Hematoxylin and eosin (HE) staining, NISSL staining, and transmission electron microscopic examination were used to evaluate alterations at the histological level. Polymerase chain reaction (PCR), Western blots, and enzyme-linked immunosorbent assays (ELISA) were employed in biochemical analysis.

**Results:**

The BBB score and inclined plane test score significantly decreased after SCI surgery, whereas chronic FA treatment (dose of 90 mg/kg, i.g.) for 28 days improved SCI-induced motor dysfunction. HE staining showed that SCI surgery induced internal spinal cord edema, but the structural changes of the spinal cord could be reversed by FA treatment. NISSL staining and transmission electron microscopic examination confirmed the improvement of the effect of FA on the injury site. In the biochemical analysis, it could be found that FA inhibitedSCI-induced mRNA and protein overexpression of pro-inflammatory cytokines (IL-1β, IL-6, TNF-α), as well as iNOS and COX-2 via the modulation of NF-κB level in the spinal cord of SCI rat. Moreover, the SCI-induced decrease of Bcl-2/Bax ratio was also reversed by FA treatment. However, the effect of FA on the expression of Beclin-1 was not statistically significant.

**Conclusions:**

FA showed a therapeutic effect on SCI, which may be associated with the regulation of neuroinflammation and apoptosis.

## Introduction

Spinal cord injury (SCI) is a devastating trauma of the central nervous system (CNS) that seriously affects the quality of one’s life^1^. The development of SCI includes primary injury and secondary injury. The primary injury usually refers to the physical injury caused by mechanical compression to the spinal cord at initial impact. Secondary injury is a series of complex pathological changes following the primary injury^2^. Since primary injury is irreversible, avoiding secondary injury becomes the key to the clinical treatments for SCI.

As it is known, trauma to the spinal cord causes immediate nervous tissue injury, which leads to acute inflammatory and destroys blood vessels in the injury epicenter, and the vascular damage exacerbates inflammation of the damaged nerve tissue. In the process of immune response after SCI, inflammatory cells, including T cells, macrophages, as well as neutrophils, rapidly migrate and infiltrate the injury site^3^. Subsequently, inflammatory cytokines such as IL-1β, IL-6, and TNF-α were overexpressed in the injury epicenter^4^^,^ 5. As tissue-resident macrophages of CNS, microglial cells are activated after pathologic stimuli and produce large amounts of prostaglandin and nitric oxide, which are largely regulated by two inducible rate-limiting enzymes, COX-2 and iNOS^6^.

Apart from inflammatory response, apoptosis and autophagy, two different programmed cell death forms, were also demonstrated to play important roles in the secondary injury stage of SCI^7^. For example, Beclin-1, which is a well-established regulator of the autophagic pathway, has been implicated to be involved in the pathology of SCI^8^. Besides, Bax and Bcl-2 are two important factors associated with neural cell apoptosis^9^^,^ 10. Therapeutic interventions modulating the aforementioned cytokines relating to inflammatory response, cell apoptosis, and cell autophagy, three major pathogeneses of neuron loss, may be beneficial to diminish the secondary injury of SCI.

Ferulic acid (FA), the main active ingredient of herbal medicine *Angelica sinensis*, possesses various pharmacological functions. For example, a previous study showed FA presented neuroprotective function against ischemia/reperfusion (I/R)-induced brain injury via suppressing oxidative stress and apoptosis^11^. Another basic research found FA protects hyperglycemia-induced kidney damage by regulating oxidative insult, inflammation, and autophagy^12^. Though the protective effect of FA on SCI has been reported in a few studies^13^^,^ 14, the underlying mechanism is largely unexplored.

The present study was designed to investigate FA’s effect on SCI by evaluating the motor function of rats before and after SCI surgery. The expressions of inflammatory-related mediators (NF-κB, IL-1β, IL-6 TNF-α, iNOS, and COX-2), apoptosis-related proteins (i.e. Bcl-2, Bax), and autophagy-related factor Beclin-1 were tested to disclose the mechanisms associating with FA’s possible positive function on SCI.

## Methods

All the experimental procedures were approved by Zhejiang Pharmaceutical College Animal Care and Use Committee (approval number: wydw2017-0051) and conducted according to the guidelines set forth by Chinese National Institutes of Health. In the experiment, five animals were lost due to SCI, and another five were used as substitutes. Thus, a total of 55 rats was used in this study.

Adult male Wistar rats (220-240 g) were purchased from Shanghai Animal Center of Chinese Academy of Science. The rats were housed five per cage under controlled environmental conditions. Once arrived, the rats adapted to the environment for one week and later were used in the experiment. Fifty rats were evenly allocated into five groups: sham (animal received surgical operation without SCI), SCI, and SCI+FA groups, the latter divided into doses of 10, 30, 90 mg/kg, i.g.

### Treatment schedule

**Figure 1:**
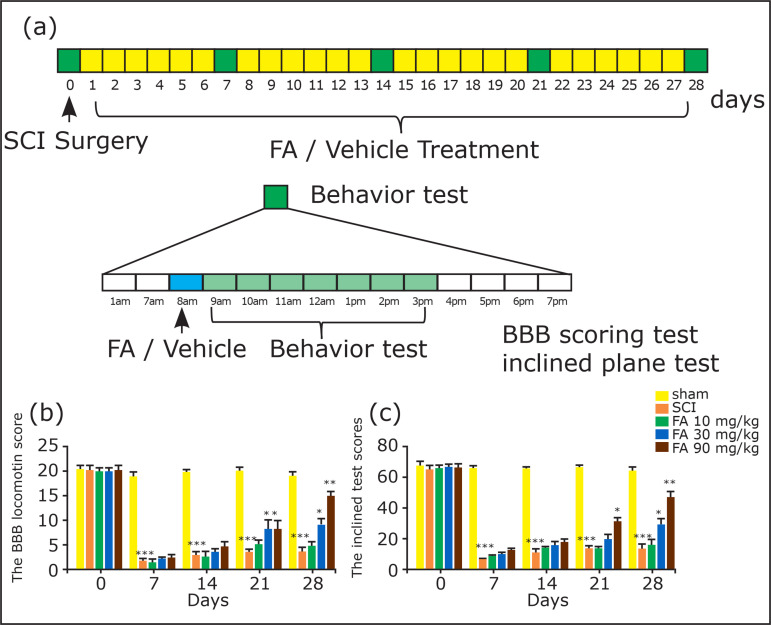
**(a)** Experimental design. SCI model was established by creating a moderate spinal cord compression using a vascular clip. The sham group received the same surgical procedures without compression generated by vascular clip. After SCI surgery, rats received FA (10, 30, 90 mg/kg, i.g.) or vehicle (CMC-Na) for 28 days. BBB scoring method and inclined plane test were performed to assess the motor function of rats on days 0, 7, 14, 21, and 28 post-surgery. Animals were sacrificed on the 28th day after behavior tests for neurochemical analysis. Effects of FA (10, 30, 90 mg/kg, i.g.) on SCI rats in **(a)** the BBB locomotion test and **(b)** inclined plane test. Data are presented as mean ± SEM, n=10 in each group. ***p<0.001 when compared with the sham group;

The rats received SCI surgery except the sham group. After the surgery, rats received FA (doses of 10, 30, 90 mg/kg, i.g.), which was dissolved by carboxymethyl cellulose sodium for 28 consecutive days (each day at 8 a.m.). The dose of FA was selected based on a previous study^15^. Each animal received a behavioral test on days 0, 7, 14, 21, and 28 post-surgery. On day 28, rats were anesthetized by pentobarbital (4 mg per 100 g body weight, i.p.). For histological experiments, rats (n=5 in each group) were perfused with saline, followed by formaldehyde phosphate buffered saline (PBS) solution. For biochemical experiments, the clean spinal cord tissues (from T8 to T10) of the rats (n=5 in each group) were retrieved after perfusion with saline. The experimental design is summarized in Fig. 1a.

### Spinal cord injury surgery

SCI surgery was performed as previously described^16^. Firstly, the rat was anesthetized by pentobarbital (4 mg per 100 g body weight, i.p.) and, then, placed in a prone position on a platform, and all four feet were fixed. Afterward, the spinal cord T9 was exposed and clipped by a vascular clip for 2 minutes (30 g forces, Oscar, China) to induce SCI. Postoperative care included bladder massage twice a day for three days and passive mobilization of hind legs three times a day.

### Motor function test

The locomotor function of rats was evaluated by BBB scoring test^16^ and inclined plane test^17^ on days 0, 7, 14, 21, and 28 after SCI. In the BBB scoring test, the activity of the hind limb of each animal was videotaped and recorded by three blinded observers. BBB scores ranging from 0 to 21 represent the state from no hind limb movement to normal gait. In the inclined plane test, the maximum angle at which the animal could maintain for 5 seconds without falling was the data of this test.

### Hematoxylin and eosin staining and NISSL staining

The retrieved spinal cord tissue was embedded in paraffin. The lesion epicenter was stained with hematoxylin and eosin (HE staining) or cresyl violet (NISSL staining), according to the standard protocols (HE Staining Kit and NISSL Staining Kit, purchased from Beijing Solarbio Science & Technology, Beijing, China). All stained sections were further observed under a light microscope (Nikon, Minato, Tokyo, Japan).

### Transmission electron microscopic examination

The retrieved spinal cord tissue was bathed in 2.5% glutaraldehyde for 2 hours. The samples were dehydrated and washed, and then post-fixed in 1% osmium tetroxide including 0.8% potassium ferrocyanide and 0.1 M cacodylate buffer containing 5 nM calcium chloride for 90 minutes. After that, the samples were dehydrated in graded acetone, infiltrated with Poly/Bed 812 resin (Polysciences, Washington, PA, United States) and polymerized for 60 hours. Five hundred-nanometer-thick sections were cut on an ultramicrotome (Leica Ultracut UCT) and stained with toluidine blue. Images were obtained using a digital camera (DP 11, Japan) attached to a microscope (Olympus Ax70).

### Quantitative real-time polymerase chain reaction

The mRNA levels of L-1β, IL-6, TNF-α, iNOS, and COX-2 in the spinal cord were measured by quantitative real-time polymerase chain reaction (qRT-PCR). Total RNA was isolated using Trizol reagent (Trizol Invitrogen) according to the manufacturer’s protocol, and RNA (1 mg) was reversely transcribed using MJ Mini Gradient Thermal Cycler (Bio-Rad Laboratories, Hercules, CA, United States). RNA concentration was determined using a spectrophotometer (Bio-Rad Laboratories) at 260 nm. Subsequently, extracted RNA was reversely transcribed into complementary deoxyribonucleic acid (cDNA) following PrimeScript RT reagent Kit (Otsu, Shiga, Japan). SYBR Green (iQ SYBR Green supermix reagent, Bio-Rad Laboratories) was added to each sample at a concentration of 50 nmol/L.

The protocol of the real-time PCR was as follows: initial denaturation at 95°C for 10 min, followed by 40 cycles at 95°C for 10 s, and 58°C for 30 s. At the end of the PCR reaction, a melting curve was obtained by holding at 95°C for 15 s, cooling to 60°C for 1 min, and then heating slowly at 0.5°C/s until 95°C. PCR products were amplified in the real-time PCR machine followed by melt curve analysis. All the data were normalized to the housekeeping gene β-actin. The primer sequences were listed in Table 1.

**Table 1:** The primer sequences of target mRNAs.

Target	Forward (5’-3’)	Reverse (5’-3’)
IL-1β	TGGACTTCGCAGCACAAAATG	GTTCACTTCACGCTCTTGGAT
IL-6	CCAGAAACCGCTATGAAGTTCCT	CACCAGCATCAGTCCCAAGA
TNF-α	GCTGGATCTTCAAAGTCGGGT GTA	TGTGAGTCTCAGCACACTTCCATC
iNOS	CCTCCTCCACCCTACCAAGT	CACCCAAAGTGCTTCAGTCA
COX-2	TGGGTGTGAAAGGAAATAAGGA	GAAGTGCTGGGCAAAGAATG
β-actin	TGGAATCCTGTGGCATCCATGAAAC	AAAACGCAGCTCAGTAACAGTCCG

Western-blot analysis

The protein levels of Bcl-2, Bax, and Beclin-1 were detected by Western-blot assay. The spinal cord tissue was incubated with RIPA lysis buffer (Millipore Chemicon, Temecula, CA, United States), and then the supernatant was obtained by centrifugation. The protein concentration of each sample was determined by BCA assay kit (Thermo Fisher Scientific, Waltham, MA, United States), and 40 μg protein was included in each band. After electrophoresis and membrane transfer, the blots were incubated with blocking buffer for 2 h, washed by washing buffer, and incubated with primary antibodies–anti-Beclin-1 1:400, purchased from Santa Cruz Biotechnology (Dallas, TX, United States); anti-Bcl-2 1:2,000, anti-bax 1:1,000, and anti-β-actin 1:1,000, purchased from Abcam Plc (Cambridge, United Kingdom). Afterward, the blots were incubated with secondary antibodies (1:10,000) and finally imaged by fluorescence scanner (Odyssey Infrared Imaging System, South San Francisco, CA, United States).

### Enzyme-linked immunosorbent assay

Expressions of NF-κBp65 and pro-inflammatory cytokines (IL-1β, IL-6, and TNF-α) in the spinal cord were tested by enzyme-linked immunosorbent assay (ELISA) kits purchased from R&D System (Minneapolis, United States). The levels of iNOS and COX-2 in the spinal cord were detected by ELISA detection kits obtained from Abcam (Shanghai, China). In the experiment, the protein standard and sample solutions were firstly added into a 96-well plate respectively, followed by the addition of the anti-antibody. After washing, a mixture containing avidin and horseradish peroxidase was added to the plate. The reaction was stopped by the terminating solution, and the optical density (OD) values of IL-1β, IL-6, TNF-α, iNOS, and COX-2 were tested by spectrophotometer at 450 nm wavelength. Only the OD value of NF-κBp65 was measured at 405 nm wavelength. The concentration of each sample was obtained by using the standard curve provided by the manufacturer based on the OD value.

### Statistical analysis

SPSS software (International Business Machines Corporation, Endicott, NY, United States) was used for data analysis. Multiple-group comparisons were analyzed by one-way analysis of variance (ANOVA). Two-group comparisons were analyzed by the Dunnett’s test. The results were presented as mean ± standard error mean (SEM), with p < 0.05 being considered as a statistical difference.

## Results

### Effects of ferulic acid on the locomotor function of rats

As shown in Fig. 1b, SCI surgery led to a significant decrease of BBB score in the SCI group when compared with the sham group (p < 0.001). However, the BBB score gradually increased with the chronic treatment of FA, especially at 90 mg/kg, and the maximal effect of FA was observed on day 28 post-surgery at the concentration of 90 mg/kg (p<0.01). Similarly, the angle of incline reduced markedly after SCI (p < 0.001) (Fig. 1C), whereas FA (90 mg/kg, i.g.) relieved this adverse effect, particularly on day 28 post-surgery (p < 0.01). Based on these behavioral results, FA was shown to protect the rats from motor dysfunction induced by SCI.

### Spinal cord histology of rats

**Figure 2:**
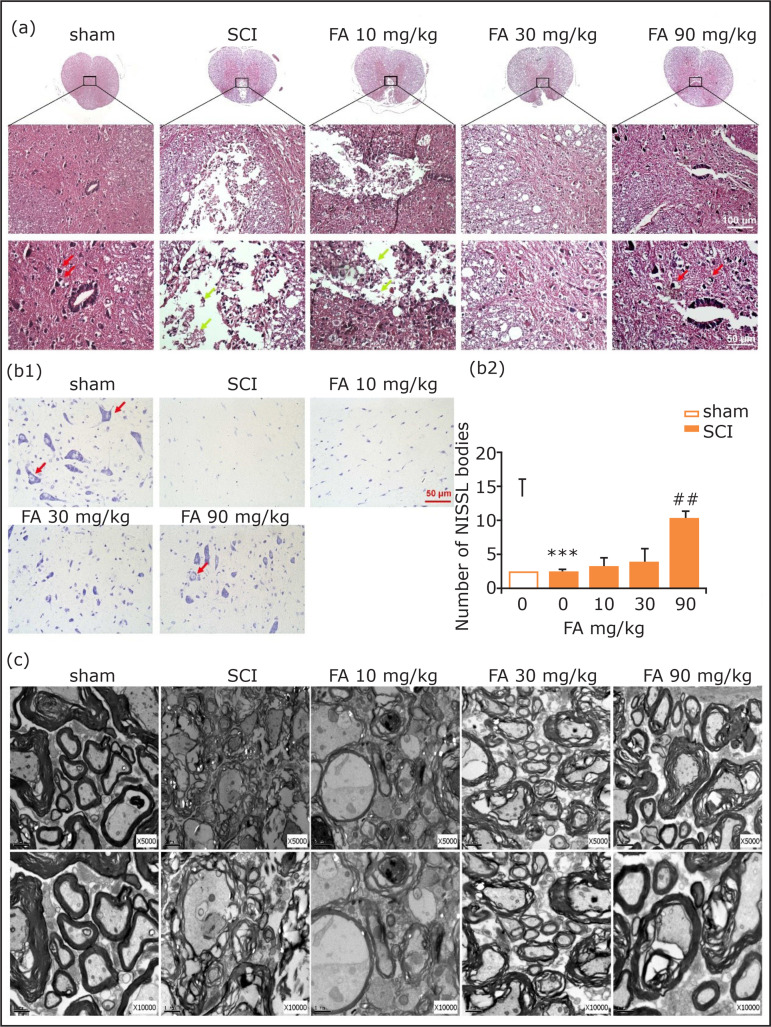
**(a)** HE staining on transverse section of the spinal cord of T9 in rats at the 28th day after spinal cord injury (the first row is ×50, the second is ×200 and the third is ×400). Red arrow: normal nerve cells, green arrow: lesion site after spinal cord injury. **(b)** NISSL staining (×40) on transverse section of spinal cord T9 in rats and quantitative analysis of NISSL bodies in staining images (n = 4 in each group), scale bar = 50 μm. Red arrow: NISSL bodies. **(c)** Ultrastructural morphology of myelin sheath and neuronal cells in the dorsal column and epicenter surrounding the gray matter of different groups. Data are presented as mean ± SEM. ***p < 0.001 when compared with sham group;

As seen in Fig. 2a, normal spinal cord neurons had clear cell outlines and cytoplasm with uniform nuclei, while in SCI rats the lesion center was characterized by the destruction of gray and white matter. The neurons in the anterior horn shrunk or had pale homogenous cytoplasm. The injured tissue was prominently repaired after 28 days of FA treatment. The repairment was manifested by the recovery of nuclei and morphology and reduction of organization air conditioning in spinal gray matter.

Similarly, in rats undergoing SCI surgery, NISSL bodies in the anterior horns were significantly decreased when compared with the sham group at the 28th day (p < 0.001, Fig. 2b). However, FA treatment reversed SCI-induced decreasing of NISSL bodies (p < 0.01). These results confirmed the neuroprotective effect of FA on SCI rats.

### Spinal cord neurons morphology of rats

To further confirm the beneficial effect of FA on SCI rats, ultrastructural analysis of the epicenter and its surrounding area was performed on day 28 post-surgery (Fig. 2c). In the sham group, nerve cells showed normal morphology, and the axons were myelinated with a compact multilayered sheath. SCI surgery-induced obvious cellular damage, including dissolved cavitation, karyopyknosis and degenerated myelin sheath with a loose state. However, FA treatment revised these phenomena, especially at the concentration of 90 mg/kg.

### Effect of ferulic acid on NF-κB expression in rats

**Figure 3:**
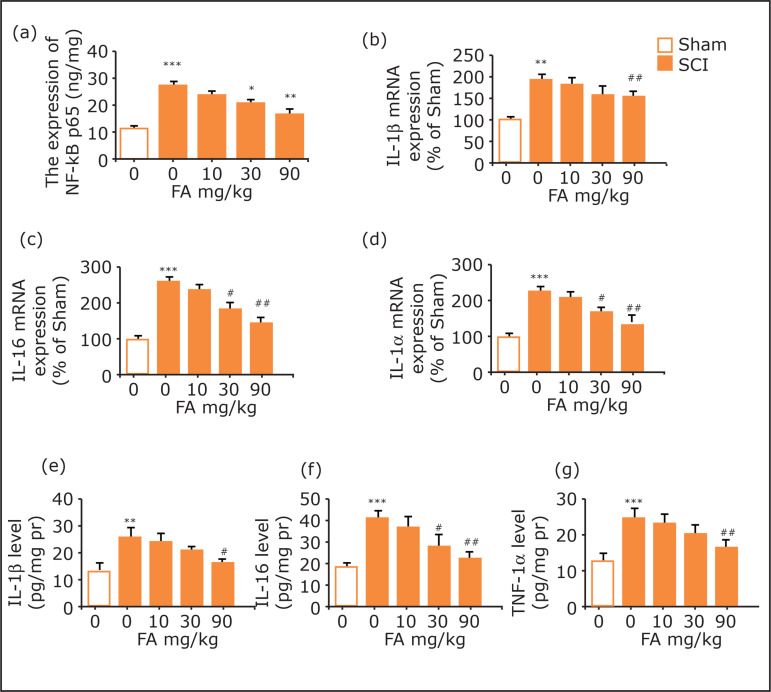
**(a)** Effects of FA on NF-κBp65 expression in the spinal cord. **(b-d)** Effects of FA on mRNA expressions of IL-1β, IL-6, and TNF-α in the spinal cord. **(e-g)** Effects of FA on expressions of IL-1β, IL-6, and TNF-αin spinal cord. Data are presented as mean ± SEM, n=5 in each group; **p<0.01 and ***p<0.001 when compared with the sham group;

As shown in Fig. 3a, the expression level of pNF-κB p65 in the spinal cord was notably enhanced after SCI (p<0.001). Treatment with FA (90 mg/kg, i.g.) suppressed SCI-increased NF-κB p65 phosphorylation level (p < 0.01).

### Effects of ferulic acid on inflammatory factors IL-1β, IL-6, TNF-α, COX-2, and iNOS expressions in rats

SCI surgery led to significant increases in the mRNA levels of IL-1β, IL-6, and TNF-α in the spinal cord (p < 0.01 for IL-1β, and p<0.001 for IL-6 and TNF-α). Nevertheless, these increases were reversed by chronic treatment with FA (p < 0.01 for IL-1β, IL-6, and TNF-α, Fig. 3b-d). Results of the ELISA assay showed significant increases of IL-1β, IL-6 and TNF-α expressions in the spinal cord after SCI (p < 0.01 for IL-1β and TNF-α, and p<0.001 for IL-6, Fig. 3e-g), whereas FA treatment reversed the increases of IL-1β, IL-6 and TNF-α (p < 0.05 for IL-1β, and p < 0.01 for IL-6 and TNF-α).

**Figure 4:**
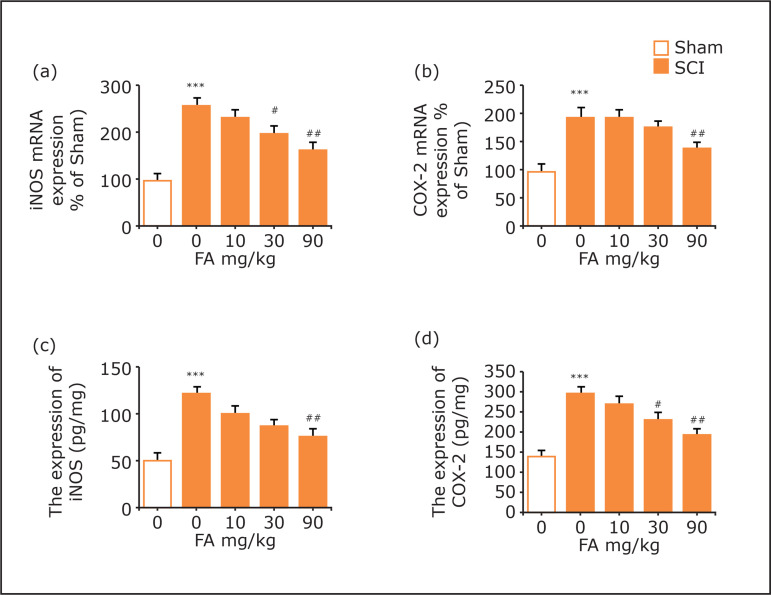
Effects of FA on mRNA expressions of **(a)** iNOS and **(b)** COX-2 in the spinal cord. Effects of FA on protein expressions of **(c)** iNOS and **(d)** COX-2 in the spinal cord. Data are presented as mean ± SEM, n = 5 in each group; **p<0.01 and ***p<0.001 when compared with sham group;

As shown in Fig. 4a-b, SCI led to significant increases in mRNA levels of iNOS and COX-2 in the spinal cord when compared with the sham group (p < 0.001 for iNOS and COX-2). Treatment with FA at 90 mg/kg markedly reversed the increased iNOS and COX-2 levels (p < 0.01 for iNOS and COX-2). A similar phenomenon could be found in the results on iNOS and COX-2 protein expressions, as illustrated in Fig. 4c-d.

### Effects of ferulic acid on Bcl-2, Bax, and Beclin-1 expressions in SCI rats

**Figure 5:**
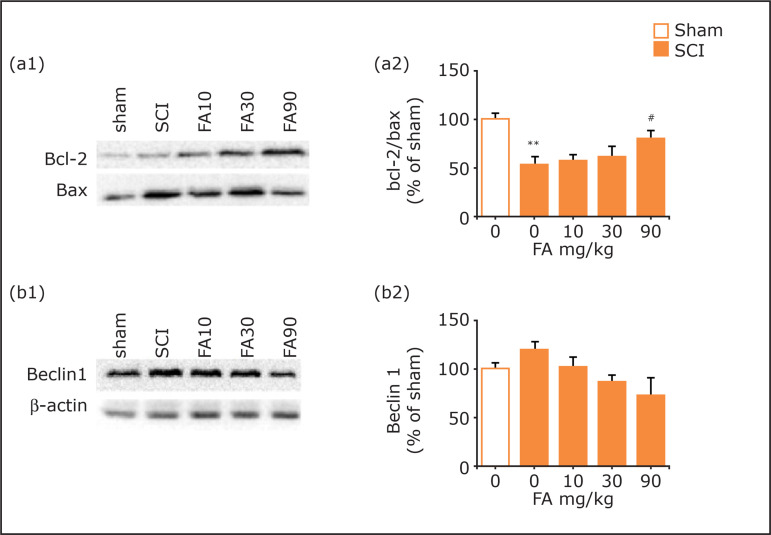
Effects of FA on **(a1-a2)** Bcl-2/Bax ratio and **(b1-b2)** Beclin-1 in the spinal cord. **(a1)** Blots of Bcl-2 and Bax; **(b1**) Blots of Beclin-1 and β-actin; **(a2-b2)** Quantitative analysis of Bcl-2, Bax, and Beclin-1 expressions in Western-blot assay. Data are presented as mean ± SEM, n = 5 in each group; **p<0.01 when compared with the sham group;

SCI surgery led to a significant decrease in the ratio of Bcl-2/Bax (p < 0.01, Fig. 5 a1-a2). FA (90 mg/kg) treatment for 28 days improved this phenomenon (p < 0.05). For Beclin-1 expression, no significant difference could be observed between different groups (Fig. 5b1-b2).

## Discussion

In our study, FA was found to have protective efficacy against motor dysfunction in SCI rats. This beneficial effect was related to the modulation of inflammatory mediators (i.e., NF-κBp65, IL-1β, IL-6, TNF-α, COX-2, and iNOS) and apoptosis-related proteins (i.e. Bcl-2, Bax) in injured nervous tissue by FA.

SCI model is a well-established animal model leading to motor dysfunction and structural lesions of the spinal cord^16^^,^ 18. In our study, SCI surgery-induced motor dysfunction, which was evidenced by significant decreases of BBB score and angle of the incline in SCI rats. The further histological evaluation indicated SCI surgery-induced structural destruction of gray and white matter. Besides, a significant decrease of NISSL bodies in SCI rats also revealed the pathologic change in the spinal cord tissue. Moreover, the spinal cord neuron damage in SCI rats was observed by ultrastructural analysis. The aforementioned results indicated that the SCI rat model in our study was successfully established.

The neuroprotective function of FA had been discovered in several previous studies^11^^,^ 19 ^-^ 21, while studies concerning the protective function of FA on SCI were very limited. In the relevant study by Wei *et al*.^14^, it was proved that the systemic function of FA combined with glycol chitosan could improve functional recovery of rats after acute SCI. In the present study, we evaluated the effect of FA on rats suffered from SCI. Behavior tests showed significant recovery of motor function in SCI rats after FA treatment. Importantly, results of histopathological examinations confirmed the neuroprotective effect of chronic FA treatment on nerve trauma in SCI rats.

Neuroinflammation is thought to play a pivotal role in the secondary injury stage after SCI^17^^,^ 18. NF-κB, the nuclear transcription factor, is a critical regulator of various pro-inflammatory cytokines^22^^,^ 23. Recently, evidence suggested that NF-κB signal pathway may be involved in the underlying neurobiological mechanism of SCI^24^. To know whether NF-κB is involved in the improvement effect of FA on SCI, we measured the expression level of NF-κB p65, which is a relevant protein involved in NF-κB heterodimer formation and nuclear translocation and activation^25^. Results indicated that NF-κB p65 was up-regulated in the spinal cord after SCI, and it was down-regulated by FA treatment, suggesting that FA’s positive function on SCI rats was achieved by inhibiting neuroinflammation through regulating NF-κB.

As downstream factors of NF-κB, IL-1β, IL-6, and TNF-α have been reported to play important roles in the development of SCI^26^^,^ 27. For example, Habgood found TNF-α was highly expressed about 30-45 min after SCI, and expressions of IL-1β and IL-6 were also significantly increased 3–24 h after SCI^4^. Moreover, TNF-α activates resident Schwann cells and accelerates macrophage recruiting to the injury site, which induced a series of inflammatory reactions^28^. In our study, IL-1β, IL-6, and TNF-α levels were significantly increased after SCI surgery, and FA treatment reversed the SCI-induced neuroinflammation.

Although FA’s anti-inflammation function has been reported in the brain, there are very few studies focusing on its anti-inflammatory effect in the spinal cord. Our data indicated that the improvement effect of FA on SCI may be due to the regulation of IL-1β, IL-6 and TNF-α through regulating NF-κB. In addition to IL-1β, IL-6, and TNF-α, COX-2 and iNOS are important roles in the pathogenesis of neurological diseases. They had been proved to remarkably increase under the regulation of NF-κB in stimulated microglial cells in rat’s brain, which constitutes the inflammatory processes^29^^,^ 30. iNOS and COX-2 were also demonstrated to be involved in the pathophysiology of SCI^31^. Suppressing of COX-2 and iNOS after spinal cord trauma has been found to exert neuroprotective effects^32^^,^ 33. Our results indicated iNOS expression was increased after SCI, and FA treatment inhibited the production of iNOS. Moreover, increased expression of COX-2 was also observed in our study, and FA reversed this phenomenon. Therefore, we hypothesize that the improvement effect of FA on SCI may also be associated with its inhibition of iNOS and COX-2 by regulating NF-κB.

In addition to the neuroinflammation mechanism, apoptosis also plays a critical role in the secondary damage of SCI^34^. Apoptosis refers to programmed cell death under precise regulation, which is executed by some evolutionarily conserved families, such as Bcl-2 and caspase families^35^. As for the Bcl-2 family, the degree of apoptosis is generally decided by the ratio of Bcl-2/Bax. Bcl-2 is an antiapoptotic member, and Bax is a pro-apoptotic protein in the Bcl-2 family. Decreased expression of Bcl-2 could leave Bax unopposed and promote apoptosis^36^. Our results showed that FA revised SCI-induced increased ratio of Bcl-2/Bax, suggesting FA’s neuroprotective function may be related to its anti-apoptotic effect.

Autophagy is another mechanism involved in the development of SCI. Via the autophagy pathway, certain toxins and pathogens are wrapped, degraded, and then eliminated^37^. Beclin-1 is an autophagy-related gene and a direct executor of autophagy^38^. In our data, Beclin-1 showed a trend of increase after SCI and decrease after FA treatment, but the result had no statistical difference. Autophagy and apoptosis are interrelated to a consistent degree. When anti-apoptotic proteins (i.e., Bcl-2, caspase) are activated, autophagy marker proteins (i.e., Beclin-1) will decrease, leading to the inhibition of autophagy^39^. On the contrary, increased anti-apoptotic proteins (i.e., Bcl-2, caspase) lead to autophagy activation^7^^,^ 40. Though a significant difference of Beclin-1 between distinct groups was not observed in our study, we speculate that the effect of FA on SCI was probably associated with inhibition of autophagy in consideration of the relationship between apoptosis and autophagy. However, more data need to be obtained in future studies to validate our speculation.

Our data revealed the beneficial effect of FA on SCI and the possible mechanisms. Despite these important findings, limitations should not be ignored. Firstly, the Beclin-1 expression only showed an increasing trend with no statistical significance, the anti-autophagy mechanism needs to be further confirmed. Second, as for the anti-inflammation and anti-apoptotic mechanisms, we only tested the expressions of several related cytokines, critical enzymes or factors involved in the pathways that had not yet been identified. Finally, the relationship between anti-inflammation, anti-apoptotic, and anti-autophagy mechanisms involved in the beneficial function of FA was not investigated, which deserves further exploration.

## Conclusions

The present study demonstrated that FA, a naturally occurring molecule, can ameliorate the motor dysfunction induced by SCI surgery in rats, indicating FA is a potential candidate for clinical SCI therapy. This positive function of FA may be achieved by suppressing neuroinflammation and apoptosis, through regulating the levels of inflammatory cytokines IL-1β, IL-6 TNF-α, iNOS, and COX-2 and apoptosis-related protein Blc-2/Bax. Further studies need to be done to investigate the specific signaling pathways involved in FA’s function on SCI.
